# Modular and Integrated Systems for Nanoparticle and Microparticle Synthesis—A Review

**DOI:** 10.3390/bios10110165

**Published:** 2020-11-03

**Authors:** Hongda Lu, Shi-Yang Tang, Guolin Yun, Haiyue Li, Yuxin Zhang, Ruirui Qiao, Weihua Li

**Affiliations:** 1 School of Mechanical, Materials, Mechatronic and Biomedical Engineering, University of Wollongong, Wollongong, NSW 2522, Australia; hl108@uowmail.edu.au (H.L.); gy417@uowmail.edu.au (G.Y.); 2 Department of Electronic, Electrical and Systems Engineering, University of Birmingham, Edgbaston, Birmingham B15 2TT, UK; YXZ048@student.bham.ac.uk; 3 Department of Chemistry and Biochemistry, University of California, San Diego, CA 92093, USA; hal412@ucsd.edu; 4 ARC Centre of Excellence in Convergent Bio-Nano Science and Technology and Australian Institute for Bioengineering and Nanotechnology, The University of Queensland, Brisbane, QLD 4072, Australia

**Keywords:** synthesis, nanoparticles, microparticles, microfluidics, integrated systems, modularisation

## Abstract

Nanoparticles (NPs) and microparticles (MPs) have been widely used in different areas of research such as materials science, energy, and biotechnology. On-demand synthesis of NPs and MPs with desired chemical and physical properties is essential for different applications. However, most of the conventional methods for producing NPs/MPs require bulky and expensive equipment, which occupies large space and generally need complex operation with dedicated expertise and labour. These limitations hinder inexperienced researchers to harness the advantages of NPs and MPs in their fields of research. When problems individual researchers accumulate, the overall interdisciplinary innovations for unleashing a wider range of directions are undermined. In recent years, modular and integrated systems are developed for resolving the ongoing dilemma. In this review, we focus on the development of modular and integrated systems that assist the production of NPs and MPs. We categorise these systems into two major groups: systems for the synthesis of (1) NPs and (2) MPs; systems for producing NPs are further divided into two sections based on top-down and bottom-up approaches. The mechanisms of each synthesis method are explained, and the properties of produced NPs/MPs are compared. Finally, we discuss existing challenges and outline the potentials for the development of modular and integrated systems.

## 1. Introduction

Particles, generally referred to as small pieces of material with sizes ranging from a few nanometres to hundreds of micrometres, possess many unique chemical and physical properties that are different from those of bulk materials. At large, particles can be categorised into two groups: nanoparticles (NPs) and microparticles (MPs) based on sizes. Owing to their large surface areas in relation to the mass, NPs and MPs are applied in areas such as catalysis [1,2,3], sensing [4,5], and imaging [6,7]. Moreover, materials in such small scale can be easily handled, transported, and processed compared with the same materials in bulk.

In general, NPs (with sizes range from a few to hundreds of nanometres) have various shapes and structures such as sphere, rod, wire, tube, cube, cone, and spiral. Besides, NPs can be crystalline or amorphous. Inorganic NPs such as nanocrystals made from noble metals or rare-earth elements can be used in drug delivery [8] and biological sensing [9]. In amorphous cases, liquid metal (LM) NPs made from gallium (Ga)-based alloys are gradually attracting attentions in fields of drug delivery, tumour therapy, and soft electronics [10,11,12]. Besides, organic polymeric nanocomposites with good biocompatibility show potentials in drug delivery vehicles [13]. The applications for various NPs are outlined and summarised in Table 1.

**Figure 1:**
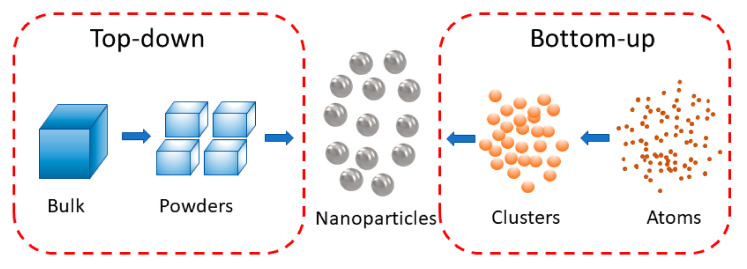
Scheme of top-down and bottom-up synthesis of nanoparticles (NPs).

A variety of synthesis approaches for NPs have been developed, and they can be categorised as either the top-down or bottom-up method, as shown in Figure 1. For the top-down approach, methods such as ultrasonication, laser ablation, and thermal decomposition have been introduced to break bulk materials into nanoscales [39,40]. In such approaches, high energy or pressure is required, and it is hard to produce NPs with sizes below 100 nm [41]. This process is also not suitable to produce uniform NPs and complex structured nanocomposites due to the uncontrollable disruptive forces. As for the bottom-up approach, NPs are synthesized by the built-up of materials from atoms to clusters to nanoscale structures. Methods such as photochemical reduction, chemical precipitation, microemulsion, microbial reduction, and hydrothermal methods are commonly used in synthesising NPs [39,41].

Both organic and inorganic MPs with sizes from a few to hundreds of micrometres have been frequently used as vehicles to carry functional molecules, such as proteins and drugs [42]. Complex MPs with customised sizes, morphologies, and compositions have been widely investigated by researchers in recent years. Preparing such MPs using conventional emulsification methods is challenging because the interfacial tension drives the automatic morphoring of emulsions into spherical shape [43]. In the past two decades, microfluidics has been adopted as a novel synthesis technique for producing MPs with customised properties [44,45]. Using monodispersed microdroplets produced by microfluidics as templates, MPs can be formed after solidification [46]. For instance, microspheres can be produced from single emulsion, while core/shell, Janus, and other irregular shapes are available from transformation of double or multiemulsions. In addition to microfluidics, millifluidic reactor (reactor with channel diameter in millimetre scales) systems have also been implemented for MPs synthesis to increase the productivity. These techniques can easily connect with typical laboratory instruments [47]. In addition, systems using acoustic [48], centrifugal [49], and jetting mechanisms [50] are developed to synthesis functional MPs. Such systems can be established easily without the need of complex microfabrication processes.

Optimising synthesis methods is beneficial in the production of quality NPs and MPs to further stimulate the development of electronic, medical, and sensing research. Despite this, most of the conventional methods require bulky and expensive equipment, leading to complex, labour-consuming operations. These limitations impede inexperienced researchers with different backgrounds to synthesise customised NPs/MPs. Additionally, without automatic control systems, most of the conventional methods follow specific protocols that require extensive experience. This lack of flexibility and controllability would require enormous and unnecessary efforts for researchers to synthesise NPs/MPs with repeatable quality and desired properties, causing inadequate utilisation of NPs/MPs in their fields of research. For example, conventional bottom-up methods produce NPs in small batches through a series of chemical reactions; the discrepancy in manual operation may cause variation of size and properties of the produced NPs from batch to batch. To address these limitations, a feasible solution is the development of “modular” and “integrated” systems for assisting the synthesis of NPs/MPs in a repeatable manner. In this solution, complex production systems are divided into various manageable modules, and the individual modules can be readily assembled and dissembled on a need base. After assembling into an integrated system, each module interacts with others and exhibits different functions. Based on the requirements of researchers, suitable modules can be chosen and assembled to produce customised NPs/MPs.

This review will discuss current modular and integrated systems for the synthesis of NPs/MPs. We categorize the systems into two main groups: systems for producing (1) NPs and (2) MPs. Systems for synthesising NPs are elucidated by top-down and bottom-up approaches. These versatile systems utilise various mechanisms such as sonication, laser ablation, microfluidics, flame synthesis, centrifugal, and spinning force for the on-demand production of NPs/MPs. Several representative examples are provided to illustrate the mechanisms and production efficiency of such systems. Finally, we envision the challenges and opportunities for the development of future modular and integrated systems.

## 2. Integrated Systems for Synthesising NPs

Numerous integrated systems have been developed to facilitate the production of NPs for research in laboratories or application in the industry without complicated operations. Here, we categorise the systems based on top-down and bottom-up methods. We first elucidate top-down production methods enabled by (1) sonication, (2) laser ablation, (3) microfluidics and millifluidics, and (4) flame synthesis. The bottom-up methods would be explained in later text.

### 2.1. Systems Based on Top-Down Methods

Synthesis mechanisms like sonication and laser ablation are suitable to produce metallic NPs for top-down methods. Bulk materials can be comminuted into nanoscales, and the existence of ligands or the rapid formation of oxide layer prevents the products from assembling back into large scales [51,52].

#### 2.1.1. Sonication Systems

**Figure 2:**
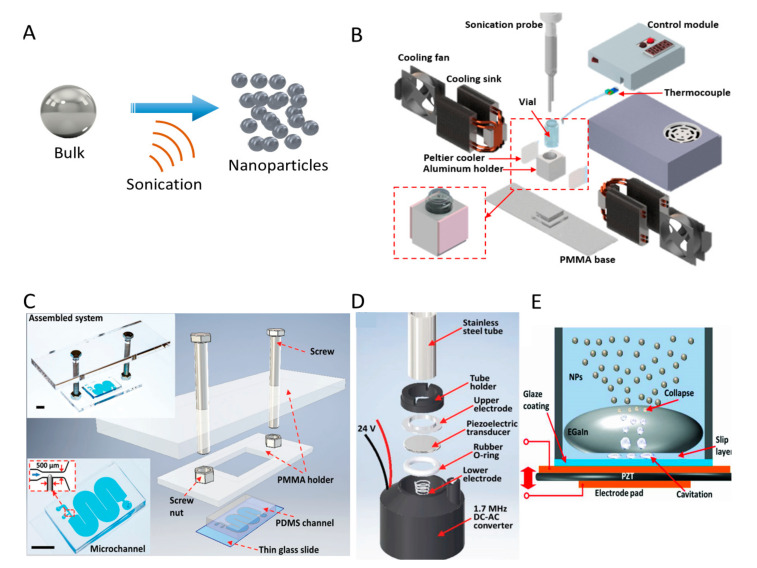
Schematics of ultrasonic mechanism and representative platforms. (**A**) Schematic of sonication mechanism. (**B**) Exploded schematic of the liquid metal (LM) NP production platform with a dynamic temperature control system. Reprinted with permission from ref [54]. Copyright (2020) American Chemical Society. (**C**) Experimental setup of the on-chip LM NP production platform. Reprinted with permission from ref [51]. (**D**) Exploded schematic of a liquid-based nebulization system. Reprinted with permission from ref [55]. (**E**) The schematic of the mechanism for producing EGaIn LM NPs.

Sonication is applied as sound energy to agitate bulk materials, as shown in Figure 2A. The sonication systems such as probe sonicator and ultrasonic bath have attracted attention for the production of LM NPs. The spontaneous formation of an oxide skin on the surface prevents LM NPs from assembling back into bulk materials. For the top-down production of LM NPs, sonication probe systems are commonly used, which can provide larger power density and yield NPs more efficiently than that of ultrasonic bath. However, the rapid heating induced by the high-power intensity of sonication probe drives undesired oxidation, dealloying and phase transitions of LM NPs [53]. To eliminate the undesired effects and efficiently produce LM NPs, a dynamic temperature control system was developed [54]. Figure 2B displays the exploded schematic of the system, combining sonication probe, cooling modules, control module, and power supply. The system adjusted the supplying power to Peltier cool pads at the same time by detecting the real-time temperature of the vial through the thermocouple to keep the vial in target temperature. This system is capable of producing NPs smaller than 50 nm, which is beneficial for applications in nanomedicine.

Despite the mentioned advantages of probe sonicator, the bulky and expensive nature limit its use. As a substitute, a microfluidic LM NP production platform using ultrasonic bath was created, as shown in Figure 2C [51]. This platform utilises microfluidic chips to generate microdroplets of LM and further breaks them into NPs in ultrasonic bath. By adjusting the dimensions of microchannels and the applied centrifugal force on the suspension, NPs with different size distributions of NPs can be collected. This simple and low-cost platform can be applied in any laboratories with inexpensive ultrasonic bath. In addition, Tang et al. designed a liquid-based nebulization system for producing LM NPs, as shown in Figure 2D. This cost-effective platform can achieve one-step production of stable and functional LM NPs [55]. The lack of severe turbulence after activating the transducer indicates that the production process is different from the case of using sonication probe systems, and the production mechanism can be explained using the cavitation hypothesis, as shown in Figure 2E. The generated vapor cavities of water within the slip layer between LM droplet and the surface of transducer collapse at the EGaIn–solution interface, which liberates EGaIn NPs into the surrounding suspending medium. Additionally, the thickness of oxide skin on the NPs can be controlled using an integrated electrochemistry system.

#### 2.1.2. Laser Ablation Systems

**Figure 3:**
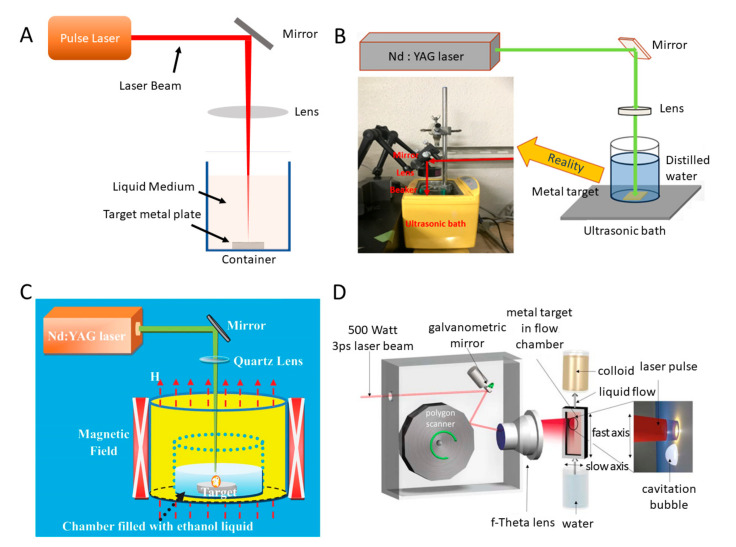
**Schematic of laser ablation systems for producing NPs.** (**A**) Schematic representation of a typical setup for liquid phase laser ablation. (**B**) An ultrasonic-assisted pulse laser ablation (PLA) system. Reprinted with permission from ref [59]. (**C**) Illustration of magnetic field assisted laser ablation system. Reprinted with permission from ref [60]. (**D**) Schematic showing the laser ablation in liquid setup. A laser beam is deflected by two scanning systems: a polygon scanner for the vertical axis and a galvanometric mirror for the horizontal axis. Reprinted with permission from ref [61] © The Optical Society.

The core of laser ablation is to ablate the surface of the solid target material by a femtosecond–nanosecond pulsed laser to synthesize NPs. It constitutes a green technique that does not need any metal precursors and reductants to stabilise the colloidal dispersions and the highly pure colloids are produced without any by-products [56]. In addition, the production process is conducted under ambient conditions without the need of extreme temperature and pressure. The typical setup of liquid-phase laser ablation is schematically presented in Figure 3A. This technique can cover a wide range of suitable target materials and solvents. By tuning laser parameters and other assisting factors, the size and shape of NPs can be controlled. Due to the formation of new phase in laser ablation involves both liquid and solid, researchers are able to select and combine various solid targets and liquids to synthesize NPs for fundamental research and industrial applications [57]. The exact mechanism is complicated and still under investigation. Generally, this method involves photon-induced material ionization, plasma phase formation, and the nucleation of NPs [58]. Due to the simplicity of processing, the production of metal NPs such as gold (Au), silver (Ag), and copper (Cu) by laser ablation is of a great scientific interest. The lack of grafting agents in general laser ablation systems causes aggregation of NPs in the solution. To fix this issue, Hu et al. established an ultrasonic-assisted pulse laser ablation (PLA) system by applying ultrasonic bath to assist the laser ablation process, resulting in a decrease in particle size and an enhanced fabrication rate, as shown in Figure 3B [59]. This system used a laser with a wavelength of 532 nm to produce Au and Ag NPs, with smaller average sizes compared with the system without an ultrasonic bath.

To efficiently produce NPs with controllable sizes, Yu et al. established a multiple-pulse picosecond fibre laser system with a 3D stage that can be adjusted to change target position while producing NPs [62]. By altering the subpulse number in an envelope, NPs with sizes ranging from 4 to 120 nm (standard error of 5%) were produced. Moreover, Mahdieh et al. utilised a Nd:YAG laser system with a Joulemeter measuring pulse energy to investigate the effects of water depth and laser pulse numbers on the size of the produced colloidal NPs including aluminium (Al) and titanium (Ti) [63]. At a certain water depth, the increase in laser pulses numbers can lead to smaller mean NP sizes. Kőrösi et al. also established a picosecond laser system for producing ligand-free size-controllable Ag NPs by applying different laser wavelengths, which can maintain stable without any additives and be applied for antimicrobial activities [64]. Herbani et al. produced Au, Ag, and Cu NPs with average sizes of 20–40 nm by a pulse laser ablation system [65]. The effective laser wavelengths for correlated NPs production are found, e.g., the effective laser wavelength for Au and Ag targets is 532 nm and that for Cu target is 1064 nm. Apart from the laser wavelength, the effects of liquid medium were also investigated using a femtosecond laser ablation-assisted system. It was determined that the deionised water medium have positive impacts on the properties of the produced Ag NPs [66].

In addition to laser and liquid medium parameters that affect the synthesis of NPs, external environment also influences laser ablation [40]. Various field-assisted laser ablation systems have been established. These fields include electric [67], magnetic [60,68,69], and electrochemical ones [70,71,72]. The germanium dioxide (GeO_2_) NPs with a high refractive index can be produced by applying different electric fields assisting laser ablation. Shapes of NPs, such as cubes or spindles, are obtained with the assistance of electric fields, in whose absence would result in spherical particles [67]. Apart from using the magnetic field to assist laser ablation, one step fabrication of one-dimensional (1D, which means a very large aspect ratio) magnetic NPs (such as cobalt carbide spheres) can be achieved, which effectively simplifies the NPs synthesis process [60]. Figure 3C illustrated the setup of the laser ablation system. During the period when laser beam is focused on the target sample, a strong and uniform magnetic field in vertical direction is applied to achieve the synthesis of NPs and one-step fabrication of 1D chains. Moreover, external magnetic field enables the acceleration of Au NPs formation [68] and can increase the aspect ratio (up to 17–18) of Au NPs [69]. Additionally, electrochemistry-assisted laser ablation can change the morphology and composition of produced NPs. The products can possess excellent optical multiabsorption [72] and magnetic [70] properties. To meet the demand of industrial production, Streubel et al. created a novel laser system that achieved a continuous ablation rate up to 4 g/h to produce a variety of metal NPs, such as Au, Ag, and Pt [61,73]. This platform consisted of a 3 ps laser system (*Amphos 500flex*), two scanning systems (a polygon scanner for the vertical (fast) axis, a galvanometric mirror for the horizontal (slow) axis), a water-filled chamber, and a liquid flow system, as shown in Figure 3D. The fast axis is controlled by the polygon scanner, which reaches speeds up to 500 m/s, while the slow axis utilises the galvanometer mirror with speeds up to 10 m/s. The liquid flow system can pump the liquid continuously to take away the generated NPs that remain in the path and shield the laser pulses. The platform adapted a repetition rate of 10 MHz to bypass the cavitation bubbles, minimizing the plasma-induced cavitation bubbles that limits the productivity of NPs.

To make it convenient for researchers to distinguish and pick up feasible systems for their researches, the integrated systems developed in recent years based on these two mechanisms are outlined and summarised in Table 2. As the costs may impede researchers conducting their academic experiments, we mark the cost of each system by asterisks to discriminate different systems. The systems including bulk expensive equipment such as laser generators, syringe pumps, high temperature, and pressure reactors are regarded as high costs and marked with high number asterisks; the systems incorporating commercially available components or modules that are easy to get are regarded as low cost and gain low number of asterisks.

### 2.2. Systems Based on Bottom-Up Methods

Because the synthesis of monodispersed NPs is challenging by top-down methods, microfluidic and flame synthesis systems have been developed based on bottom-up mechanisms. By precisely controlling the crucial reaction parameters, micro-/millifluidic systems enable the production of a wide variety of organic and inorganic NPs [44], and some scalable architectures that combine individual units together in parallel. This technique promotes the potential in industrial production level [91]. In addition, without complex steps, flame synthesis systems are promising and capable of producing high-purity products like Ag [92] and carbon [93]. These two methods will be elaborated in detail in the following sections.

#### 2.2.1. Microfluidic and Millifluidic Systems

The concept of microfluidics emerged in the beginning of the 1980s. This technology can precisely control the fluid in channels on a scale of tens to hundreds of micrometres and with the features of small volumes, small size, low energy consumption, and microdomain effects. It has been widely used in engineering, physics, chemistry, and biotechnology. Regarding using microfluidic chips as microreactors, the systems can realize the synthesis of NPs, nanoframeworks, and nanocrystals. Additionally, millifluidic systems have also been implemented for NPs synthesis, achieving high production rate.

**Figure 4:**
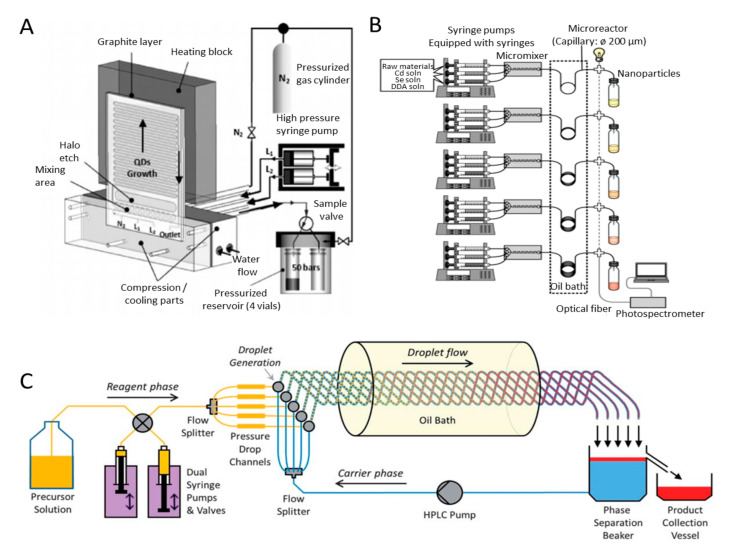
Microfluidic/millifluidic systems for producing NPs. (**A**) microfluidic system for producing CdSe quantum dots (QDs). Reprinted with permission from ref [94]. (**B**) A combinatorial synthesis system contains several microreactors for CdSe NPs production [95]. (**C**) A multichannel droplet microfluidic reactor. Reprinted with permission from ref [91].

Semiconductor NPs such as cadmium selenide (CdSe) quantum dots (QDs) with highly crystalline and narrow size distributions have been applied in biological imaging. To synthesise CdSe QDs with high quality in good control, Marre et al. designed a microfluidic system to achieve supercritical continuous-microflow synthesis process, as shown in Figure 4A [94]. This system consists of a high-pressure, high-temperature microreactor, a compression-cooling aluminium part, a high-pressure syringe pump, a 5-way high-pressure valve, and a high-pressure reservoir containing four vials. To improve the syntheses process in microreactors, high pressure is applied to the whole system. The microreactor was pressurised first from inlet to outlet by pressurised gas cylinder. Then, the two high-pressure syringe pumps assured the flow rate of precursor is controllable. The precursor in the high pressure microreactor remained liquid or become supercritical, leading to fast mixing and narrow residence-time distribution. With this system, quantum dots with different size distributions and nuclei concentrations were synthesized by adjusting the precursors initial concentration, temperature, and residence time in the microreactor. This system verified the possibility of materials synthesis in supercritical media in a continuous style at the microscale. Additionally, Toyota et al. presented a combinatorial synthesis system that contains several microreactors with high reproducibility to efficiently obtain high-performance CdSe NPs, as shown in Figure 4B [95]. This system has four sections: syringe pump, mixing section, heated reaction section, and spectroscopic measurement section. Applying the parallel operation of microreactors, the optimum sets of five reaction parameters, including the temperature, linear velocity, capillary length, reaction time, and reaction additive concentration, can be obtained to produce NPs with desired properties. The whole processing time for synthesising one batch of NPs, including raw material solutions preparation, system setup, reaction, production measurement, analysis, and instruments clean-up, is just around 20 min.

Scale-up synthesis of CdSe nanocrystals, which can meet the demand of industry production, is admirable. However, it is still challenging to develop scalable architecture that allows multiple channels to be supplied from a small number of feed reservoirs. Crucially, individual channels must be stable for operation and keep operations over extended periods of time. Nightingale et al. reported a multichannel droplet microfluidic reactor for achieving large-scale synthesis of nanocrystals [91]. A common set of feed reservoirs, two pumping systems, and five-way passive flow-drivers were contained in this platform, as shown in Figure 4C. A high-pressure liquid chromatography pump that withdraws carrier fluid can maintain a continuous flow. By adjusting back-pressure and pressure drop channels to regulate the reagent phase, the system enables the stable balanced flow distribution, providing the possibility of high yield synthesis of NPs. This system can yield 1.5 g of CdSe in the first hour, while cadmium telluride (CdTe) and cadmium selenide tellurium (CdSeTe) can be synthesized with the yields of 3.7 and 2.1 g, respectively. Moreover, the system can work a maximum of 9 h continuously to produce 145 g of CdTe and showed no signs of degeneration of performance during the running time.

However, commercial applications with Cd-based NPs are limited because of its high toxicity. Cd-free alternatives, such as indium phosphide (InP) QDs, have been of booming interest for research and industry areas. A millifluidic reactor system was designed for multistep and continuous synthesis of InP/stilleite (ZnSeS) NPs by Vikram et al. [96]. The reason of choosing millifluidic reactors instead of microfluidic reactors is the ability of producing high-quality NPs at high production rate for large-scale synthesis. The modular system included two or more distinct reactors, for core formation, shell growth, and cooling modules, and one or more inline mixing units, for adequate and fast mixing the precursors outside the reactors. A static mixer was incorporated inside the reactor modules to minimize the residence time distribution of the reactants in the reactor as it broke up the parabolic flow profile. This system yields NPs with narrow size distribution and desired optical properties by precise control over rapid and uniform heating, cooling, and mixing of flows at a high production rate. It can be further optimised by adding other modules for crystal growth kinetics studies. Additionally, the real-time observation of nucleation and the growth of lead sulphide (PbS) QDs at high temperature was first achieved in microfluidic systems by Lignos et al. [97]. The droplet-based microfluidic platform was integrated with online absorbance and fluorescence detection (collecting the real-time data during the synthesis process) for kinetic analysis of PbS QDs synthesis. More importantly, this system can be used to study the fast kinetics of nanomaterial syntheses and ion exchange reactions (e.g., the time of the nucleation of PbS occurred is <1 s).

Apart from semiconductor NPs, noble metals including Au, Ag, and platinum (Pt) group metals with different sizes and shapes in nanoscale exhibit different optical properties, which have shown potential in applications of sensing and catalysis [98]. Duraiswamy et al. presented the synthesis of anisotropic Au nanocrystal dispersion with a droplet-based microfluidic method with T-junction [99]. However, this system failed to enable a fully continuous process and limit the scaling up synthesis, as it lacked real-time monitoring for the synthesis of Au NP seeds and had limited residence time of growing nanocrystals on-chip. Lohse et al. developed a simple millifluidic benchtop reactor system for high-throughput synthesis to obtain Au NPs with different sizes and shapes [100]. This system included multiple commercially available modular components and peristaltic pumps, which can be used in almost any chemistry research laboratory. Flow rates between 35.0 and 60 mL/min for the system can produce monodisperse Au NPs. The reason for using millifluidic reactors is that they have the advantages of easy fabrication, good resistance to fouling, and the possibility for large-scale synthesis. To achieve scaling up synthesis of Au NPs, a new approach based on millilitre-sized droplet reactors was displayed by Zhang et al. [101]. Commercially available components such as syringe pump, T-connectors, and polytetrafluoroethylene (PTFE) tubes were assembled into the device. Au, palladium (Pd), and Pd-M (M = Au, Pt, and Ag) nanocrystals with controllable sizes, shapes, compositions, and structures on a yield of 1–10 g per hour have been synthesized successfully without losing uniformity, providing exciting opportunities to biomedical and petroleum industries. Cattaneo et al. utilised different setup of millifluidic reactors to synthesize monometallic Au, bimetallic AuPd, and Au@Pd core–shell NPs in continuous flow style [102]. They produced titanium dioxide (TiO2)-supported bimetallic AuPd with a mean particle size of ~2 nm, and the size distribution is narrower compared to the distribution of conventional batch methods.

**Figure 5:**
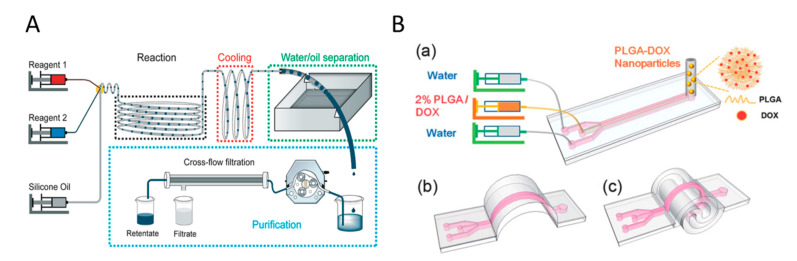
Schematic of modular microfluidic systems for producing NPs. (**A**) A droplet-reactor system with the potential for automation. Reprinted with permission from ref [105]. (**B**) A microfluidic origami chip with different configurations for enhancing mixing. Reprinted with permission from ref [106].

For synthesising Ag NPs, Matthias et al. described a microfluidic system by combining a split-and-recombine-mixer, a T-shaped mixer and a Dean-flow-mixer, to synthesize uniform seed particles for forming anisotropic Ag triangles [103]. In comparison with batch methods, the size, edge length and thickness of the seed particles can be adjusted precisely. Likewise, Cheol et al. developed a customised microfluidic reactor by assembling single functional microfluidic units, which overcame the limitations of the device reconfiguration in conventional polydimethylsiloxane (PDMS)-based microfluidic platforms [104]. The sizes of the Ag NPs can be adjusted from 4.3 to 11.45 nm with different flow rates. Since the reactor was easy to assemble and the size of the Ag NPs was controlled precisely by simply changing the injection flow rate of the precursor, the reactor can be suitable for the synthesis of NPs requiring precise control of temperature and chemical concentrations. To synthesize various noble metal NPs, Niu et al. designed a droplet-reactor system with the capabilities of both water and oil separation and the product purification, as shown in Figure 5A [105]. By Niu’s method, noble metal NPs with uniform sizes and well-controlled shapes can be easily obtained through assembling four different modules: reaction, cooling, water/oil separation, and purification. Such a droplet-reactor system is possible for automated operation by assembling the modules of both water and oil separation and product purification into a millifluidic system to further achieve continuous and scalable production of noble metal NPs in industrial scale.

Besides noble metal NPs, polymeric NPs with different functionalities provide the potential in biomedical applications. Poly(lactic-co-glycolic acid) (PLGA) NPs with great biocompatibility and biodegradability are applicable for making drug delivery vehicles, which can reduce cell toxicity and improve therapeutic efficacy [106]. To realize the best cellular uptake and anticancer efficacy, NPs with a diameter of ~100 nm are desirable. Jiashu et al. presented a microfluidic origami chip for the synthesis of PLGA NPs [106]. The novel 3D origami structure can avoid rapid velocity changes (Figure 5B), shorten the mixing distance, and reduce the mixing time, as it has smooth turns and two counter-rotating cortices perpendicular to the flow direction. Applying the novel chip, the doxorubicin-loaded PLGA NPs, which were around 100 nm with a monodisperse size distribution, were synthesized efficiently. The productivity of NPs can reach 1200 mg per day. After this work, Liu et al. designed a versatile and robust microfluidic platform with glass capillaries, which took advantage of chemical resistance [107]. The platform adopted a coflow capillary setup in which a tapered glass capillary was inserted into another bigger cylindrical one. This platform enables high linear velocity ratio between the inner and outer fluids, resulting in the fast mixing and uniform self-assembly of NP precursors with controlled microvortices and unsteady jetting. This platform can synthesize homogeneous NPs with a productivity of 242.8 g per day, a much higher than that of the origami one. Regardless of the type of organic solvents or stabilizers used, NPs with a narrow size distribution can be obtained. To further scale up the production rate and achieve precise control over the NP structure, a superfast sequential nanoprecipitation microfluidic platform was designed [108]. The platform consisted of three nested cylindrical glass capillary tubes assembled in the coaxial arrangement. High-throughput production of core/shell nanocomposites was demonstrated. By sequentially mixing nanocomposites in superfast time intervals (in microseconds range), the nanoprecipitation processes can produce stable nanocomposite cores without any stabilizers. When operated in the continuous mode, this platform can offer a high production rate of drug nanocrystal encapsulated nanocomposites up to 700 g per day, meeting the standard of clinical studies and industry production.

#### 2.2.2. Flame Synthesis Systems

**Figure 6:**
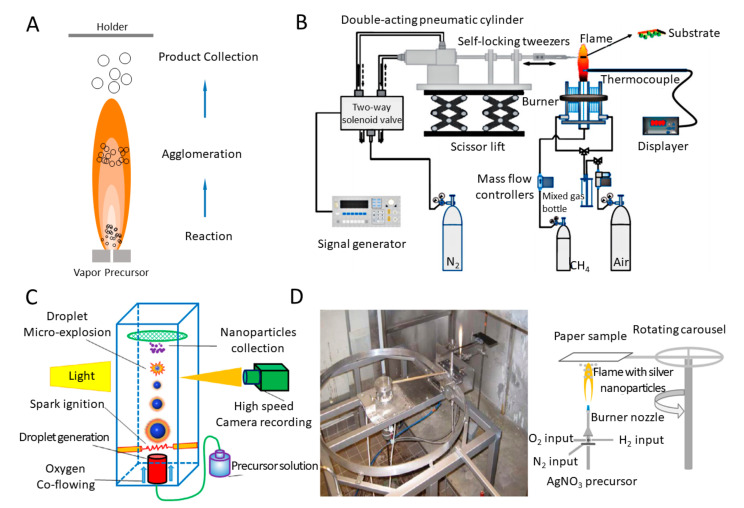
Flame synthesis systems for producing NPs. (**A**) Schematic representation of a typical flame synthesis system. (**B**) Schematic of methane coaxial jet diffusion flam system. Reprinted with permission from ref [111]. (**C**) Experimental setup for single isolated droplet combustion. Reprinted with permission from ref [112]. (**D**) The setup and schematic of the liquid flame spray (LFS) system. Reprinted with permission from ref [92].

Combustion is of great interest for material synthesis like carbon and Ag NPs for its scalability. Such a technique does not need tedious steps and can easily form high-purity products with metastable compositions [109]. The typical schematic of flame aerosol synthesis is outlined in Figure 6A. The energy from the flame flows to precursor materials to cause evaporation and drives the reaction of nucleation, agglomeration, and growth, and then, NPs formed are collected by a holder. In terms of synthesising inorganic nonmetallic NPs, Mädler et al. designed a spray apparatus consisting of an external-mixing gas-assisted nozzle, syringe pump, a glass microfiber filter, a water-cooled holder, and a vacuum pump [110]. By creating gas flow using vacuum pumps, the particles can deposit on the filter during flame spray. This system synthesized nanostructured silica (SiO_2_) particles with specific surface area using different oxidant flow rates and precursor/fuel compositions. Moreover, SiO_2_ production rate can reach up to 9 g/h (diameter of 7–40 nm) using a liquid mixture of hexamethyldisiloxane (HMDSO)/ethanol at a molar ratio of 1:10.

Mohapatra et al. was able to simplify the process of the flame synthesis and introduced a facile wick-and-oil flame system to synthesize high-quality hydrophilic onion-like carbon NPs [93]. Without any complex instruments and catalyst, the NPs produced by such system have high purity and narrow size distribution for 25 nm with a standard derivation of 5 nm. Besides, Esmeryan et al. designed a novel system that contains conical chimney with adjustable air-inlet opening [113]. They obtained amorphous, graphitic-like and diamond-like phases of carbon coatings by changing the size of opening. To dig into the effect of sampling substrate, time, and height, a methane coaxial jet diffusion flame system combines a carbon nanomaterial collection system, a burner, a flow control device, and a gas distribution system together [111]. The collection system includes a scissor lift to adjust the sampling height and a double-acting pneumatic cylinder operated by signal generator. The system can be adjusted to change the sampling time by controlling the motion of substrate in horizon direction and the tip of self-locking tweezers attached to replaceable substrate, as shown in Figure 6B. Compared with substrates of copper and nickel-chromium, carbon nanotubes collected at nickel foam substrate are produced with highest yields.

Apart from inorganic nonmetallic NPs, the synthesis of metal NPs also get benefits from the flame spray pyrolysis (FSP) system. Various integrated synthesis systems have been established on demand. For example, Li et al. established a single droplet combustion system for synthesizing tin dioxide (SnO_2_) NPs in order to provide fundamental information for the process of FSP [112]. This system combined a droplet-on demand generator, a spark ignition module, and carbon-coated Cu grids. Droplets ejected from the generator are ignited by spark from two electrodes and the formed NPs are collected on carbon-coated Cu grids, as shown in Figure 6C. Two paths of NPs formation were found, in which large SnO_2_ NPs are formed during the period for microexplosions of droplet surface, while the small ones are obtained during the steady combustion. Additionally, Brobbey et al. utilised a one-step flame synthesis method with liquid flame spray (LFS) system to produce homogeneous monolayer of Ag NPs on a paper surface [92]. Figure 6D illustrates the process by which a burner nozzle generates flame with Ag NPs, which are deposited on a paper with a rotating carousel that adjusted the passing times and speed. This environment-friendly method can deposit 30 nm NPs without effluents, and the amount of the produced NPs can be effectively adjusted by changing the passing times, which highlights potential in industry production by roll-to-roll processing.

Additionally, alumina (Al_2_O_3_) NPs can be synthesized by in-flight oxidation of flame synthesis [114]. This system adopted flames, which flow in the horizontal direction, with different ratios of oxygen and acetyl. Micro Al powders as precursors were dropped from the top, generating various sizes of NPs collected in the powder collector. Moreover, Fe ions have impacts on the synthesis and characterizations of Al_2_O_3_ NPs in the FSP system [115]. In this system, the reaction occurred in the combustion chamber and the final NPs can be collected in the microfiber filters via a vacuum pump. Different from commercial Al powders as precursors, this system used Al acetylacetonate and Ferrocene. Researchers found that two types of Al_2_O_3_ (θ- and η-) were obtained and the ability of fluoride removal is most efficient in the absence of iron, and the ferrocene added was capable of suppressing the formation of θ-Al_2_O_3_. Highly crystalline hexagonal caesium tungsten bronze (Cs_0.32_WO_3_) NPs are available by FSP followed by annealing [116]. The system integrated an ultrasonic nebulizer, to form droplets and avoid precipitation; a glass flame reactor; and a bag filter to collect NPs. The particles gained from the system were then annealed inside a tubular furnace and high-purity Cs_0.32_WO_3_ NPs were obtained after cooling naturally. Compared with conventional methods, such a system requires shorter reaction time and has high energy efficiency.

The representative integrated systems using milli-/microfluidics and flame synthesis systems are summarised in Table 3.

## 3. Integrated Systems for MPs Production

MPs with specific size and composition exhibit a promising potential in numerous areas of applications such as adductive manufacturing [126], eco-friendly electronic systems [127], and drug delivery systems [128]. Apart from traditional synthesis methods of MPs, which require redundant process and complex operation, microfluidics has been adopted as a novel synthesis technique due to its advantages of low volume and high controllability [43]. Besides, off-chip generation platforms are desirable because they are easy to operate, which facilitate inexperienced researchers [129]. Certain systems show unique advantages as they can produce MPs with a certain size range or reduce the cost of producing a certain type of MPs. For example, magnetic droplets with a diameter of ~100 μm can be simply produced by magnetically driven step emulsion device [130]. Off-the-shelf self-setting rubber can be introduced in a flow-focusing microfluidic system for the efficient and low-cost synthesis of W/O emulsions with a large size range (from 100 to 500 μm) by simply adjusting the diameter of the rubber nozzle [131]. Furthermore, some systems can achieve the versatile production of various MPs, such as LM, hydrogel, and double emulsion [129]. Based on the different production mechanisms, this section will discuss integrated systems utilising techniques including: (1) microfluidics, (2) acoustics, (3) centrifugal and spinning force, and (4) jetting.

### 3.1. Microfluidic Systems

**Figure 7:**
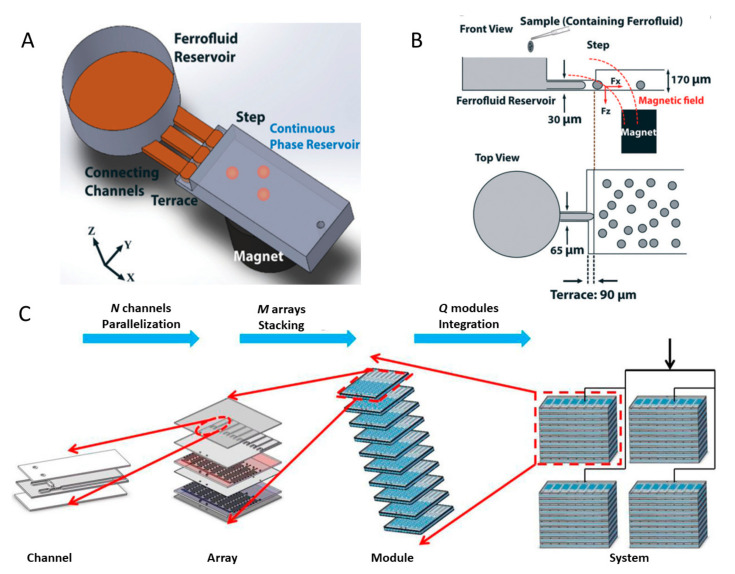
Microfluidic systems for producing microparticles (MPs). (**A**) Schematic view of a magnetically driven microfluidic droplet generation technique using ferrofluids (without any pumps). (**B**) Top and front views of the microfluidic device with dimensions. Reprinted with permission from ref [130]. (**C**) Overview of multidimensional scale-up strategy (not to scale). Reprinted with permission from ref [134].

Microfluidics is capable of producing microdroplets with specific properties by controlling and manipulating the microscale fluid in a small chip [44]. Microfluidic platforms that consists of a microfluidic chip and syringe pump can produce a variety of microspheres in one step, such as poly(3,4-ethylenedioxythiophene) (PEDOT)-based microspheres [127], enzyme-immobilised reusable polymerised microcapsules [132], and biodegradable chitosan microspheres [133]. The microfluidic chips fabrication usually requires cleanroom facilities and photolithography, impeding research carried out in laboratories without such conditions. To fabricate easy-to-make fluidic devices for producing MPs, Lapierre et al. designed a pipette tip based on microfluidic system using commercially available self-setting rubber and conventional components [131]. Such a microfluidic device can be fabricated by combining a micropipette tip with tubing in the appropriate materials and size. The entire assembly process takes less than 10 min, without any microfabrication expertise requirement. The droplet size is determined by nozzle diameter and the flow rate of each phase. Monodisperse emulsion with the size ranging from 100 to 500 µm can be generated by simply using nozzles with different inner diameters, while double water-in-oil-in-water (W/O/W) emulsions can be generated by regulating the flow rates of the three phases. The produced emulsions can be used in tissue engineering, 3D cell culture, biocatalysis, and hydrogen storage materials. In addition, Kahkeshani et al. developed a novel step emulsification device, which is driven by magnets to emulsify ferrofluids [130]. Without using pumps, the system only needs magnetic field to control the emulsification of ferrofluid-containing solutions. The device consists of a sample reservoir, connecting channels, terrace, step, continuous phase reservoir, and magnet, as shown in Figure 7A. Detailed configurations of the platform is given in Figure 7B. The magnetic field breaks the bulk ferrofluid within the emulsification device and the droplet size depends primarily on the channel geometry instead of the flow rates of the ferrofluid, interfacial forces, or magnetic force. Moreover, the droplet generation rate can be adjusted by changing the movement frequency of the magnet and the number of connecting channels between the ferrofluid reservoir and continuous-phase reservoir. This pumpless, novel method could be beneficial for point-of-care assays and cell sorting in droplets.

Apart from one-step fabrication methods with microfluidic flow-focusing systems, high throughput and scaling up productions are also widely investigated in recent years. Amstad et al. presented a microfluidic device named “millipede device.” The core of this new design is to increase the number of individual nozzles in parallel [135]. The device can produce monodisperse droplets with a diameter ranging from 20 to 160 µm and a coefficient of variations (CV) of 3%. With 550 individual nozzles in parallel, the throughput reached up to 150 mL per hour. If the millipede device is packed into array, 800 L of 160 µm droplets can be produced in the dripping regime, which provides potential in industrial applications. Kim et al. [133] presented a 512-microchannel geometrical passive breakup device was presented. Microspheres with diameters of 40.0 ± 2.2 µm can be produced (3 mL/h). The same group further improved this design by adding 256 T-junctions in the last branch of the device [136], and the micro water droplets produced can have a smaller size of 35.29 µm (CV of 8.8%) at a higher production rate of 10 mL/h. To enhance the reusability, a glass microfluidic device was designed to achieve high-throughput step emulsification with 364 linearly parallelised droplet makers and the throughput can reach up to 25 mL/h [137].

Additionally, integrated systems that combines the microchannel and different modules are developed to generate NPs with higher yield. Han et al. proposed the concept of factory-on-chip [134]. They established a large-scale 400-microchannel high-throughput system by assembling a 3D microfluidic network from channel to system. In this type of device, multiple microchannels are lined in parallel on a two-dimensional array, and multiple arrays are stacked in a reconfigurable manner in the third dimension as a module. The integration of Q modules could achieve a production rate proportional to three scale-up numbers N, M, and Q, as shown in Figure 7C. The configuration of circular array causes smaller pressure imbalance among the channels than the parallel array under the same operation conditions. Gravity and flow resistance lead to the stacking effect (i.e., the produced NPs diameters vary among different array layers) as the arrays are stacked in the vertical direction. Applying the synthesis system based on the scale-up strategy mentioned above, chitosan/TiO_2_ functional MPs with a diameter of 539.65 µm (CV of 3.59%) were synthesized in a large throughput (80 mL/min). Mohamed et al. developed a continuous and integrated microfluidic platform to produce monodisperse cell-laden microgel droplets [138]. Three main functional modules including a flow-focusing junction, an on-chip photo cross-linking chamber, and a hydrodynamic filter unit were assembled into the system. With these modules, droplets generation, separation, and other required processes can be successfully achieved. Goff et al. designed a bench-top contact flow lithography system that consists of an illumination unit, a stage unit, and an imaging unit [139]. By applying a multichannel microfluidic chip to contact flow-lithography, the platform dramatically increased the synthesis rate for chemically homogenous particles (>10^6^ particles per hour). Likewise, Jeyhani et al. developed a microfluidic flow focusing device with microneedles, which are commercially available [128]. The microneedle inserted into the microchannel formed a 3D focusing configuration that can remove the wetting effects of the disperse phase. The 3D flow focusing system enabled the control production of microdroplets with diameters ranging from 5 to 65 µm. The generation of water-in-water microdroplets can reach a production rate up to 850 Hz. To produce MPs containing radiotracers, Jia et al. for the first time designed an integrated system containing an automated radionuclide concentrator and an automated microdroplet synthesis platform. This system made possible the synthesis of MPS for patient doses of [^18^F] fluoride [140].

Furthermore, control modules that aims at fabricating monodisperse or specific-shaped MPs have been introduced to some microfluidic systems. For microdroplets produced by the conventional T-junction microfluidic channels, Zeng et al. compared two different driven methods using syringe pump and pressure pump [141]. Pressure-driven method exhibited lower polydispersity than the other one, providing theoretical support for the design of complex flow-control microfluidic systems. Furthermore, the mathematical model relating pressure fluctuations was established, which indicates that an increase in microchannel height and a decrease in width can reduce the effect of pressure fluctuations on MPs production when using T-junction microfluidic channels [142]. Crawford et al. combined imaging-based feedback and pressure-driven pumping to develop an imaged-based closed-loop feedback system for producing microdroplets with specific sizes [143]. This was achieved by acquiring volume parameters using a high-speed camera and by utilising the proportional-integral-derivative (PID) algorithm to control both the volume and frequency of droplet production. Other than imaging-based methods, Fu et al. presented an electrical-detection droplet microfluidic closed-loop control system, which could increase the efficiency and accuracy of the measurement of the droplet size [144]. Furthermore, artificial neural network (ANN) displays the potential for microdroplet prediction, and Mahdi et al. presented an ANN-based microfluidic system [145]. Two dimensionless numbers (Reynolds number and capillary number in continuous and dispersed phase, respectively; total four parameters) are picked as four neurons for input layer. The neural network architecture has 10 neurons in the hidden layer, and the dimensionless length of the drops is selected as the neuron for the output layer. Overall, 742 experiments were conducted to build up a database for the network, and the platform was capable of predicting droplet size in a high precision (mean square error is ~1.4 × 10^−6^).

**Figure 8:**
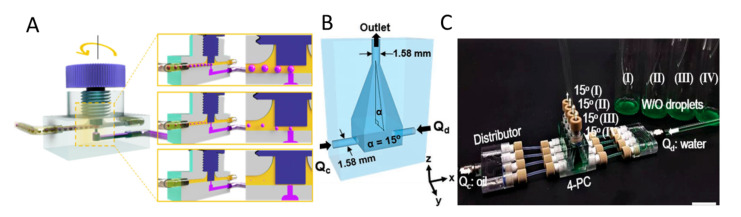
Microfluidic systems applying 3D printing technology. (**A**) Setup of a 3D-printed screw-and-nut droplet generator, the schematic also illustrates the control of the droplet size. Reprinted with permission from ref [147]. (**B**) Virtual object photographs of the 3D-printed millifluidic device with two inlets for continuous and dispersed phase and one outlet. Reprinted with permission from ref [148]. (**C**) Real image of the four-parallelised-chimneys device with the same apex angle. Scale bar is 2 cm.

Moreover, 3D-printing has been regarded as a novel technology being low-cost and convenient process and can, therefore, be applied to fabricate microfluidic devices. After assembling the device together, different types of droplets with desirable sizes can be obtained. Zhang et al. reported a 3D-printed “plug-and-play” device consisting of a generator, a commercial tubing and a fingertight fitting for generating microdroplets with a size of ~50 µm [146]. Figure 8A illustrates a 3D-printed screw-and-nut droplet generator [147]. The nut was attached vertically to T-junction droplet channels, while the screw with an external thread (total 75 teeth) was used to adjust the height of the channel. By simply rotating the screw along the axial, the gap height is adjusted between 0 and 75 μm, precisely controlling droplets with various diameters generation. The size of the produced droplets ranged from 39.0 ± 2.6 to 1404.3 ± 23.3 µm. The water-in-oil (W/O) and oil-in-water (O/W) emulsions provide the application in chemical reactions, drug testing, chemical and biological analyses, and medical engineering. To scale up production, Hwang et al. developed a 3D-printed chimney-shaped millifluidic device for producing droplets with diameters ranging from 36 to 616 µm (CV < 4%) [148]. As shown in Figure 8B, two inlets in the opposite direction at the low position of the device side allow the fluid to flow into the tapered void chamber and form droplets in a parallelised manner, which reduces the overlapping of flow channels. One outlet at the top of the chamber is responsible for transferring out the produced droplets. The control of the droplet size was achieved by adjusting the flow rate ratio between the dispersed aqueous phase and the continuous oil phase, as well as the apex angle. Moreover, the possibility of scaling up production was investigated by fabricating four chimneys with the same dimensions and incorporating them into an integrated device (4-PC); a distributer connected to the device was built to ensure the uniform supply and distribution of both the dispersed and continuous phase into the device, as shown in Figure 8C. The result showed that uniform droplets (CV less than 5%) were generated smoothly, indicating that the device can be further modularised to meet different demands and achieve scaling-up production. Furthermore, magnetic LM droplets with controllable sizes can be generated by a 3D-printed coaxial microfluidic device [149]. The device integrated three modules to produce magnetic LM droplets with diameters ranging from 650 to 1900 µm (CV < 5%) by changing the flow rate ratio of the two phases of magnetic liquid metal and poly(ethylene glycol) (PEG) solution.

### 3.2. Acoustic Systems

**Figure 9:**
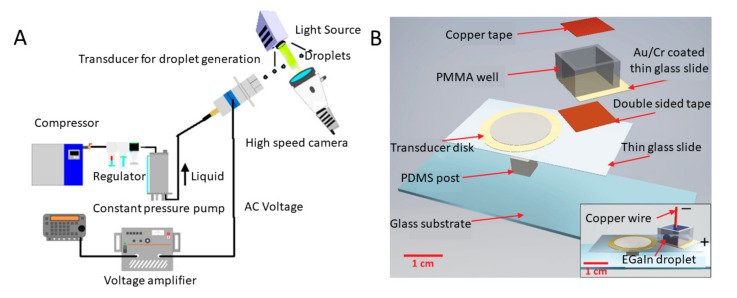
Schematics of acoustic systems for MPs production. (**A**) Schematic of a droplet generation system incorporating an ultrasonic torsional transducer and a micropore plate. Reprinted with permission from ref [150]. (**B**) Exploded schematic of the acoustic-based LM microdroplet production platform. The inset is the assembled view. Reprinted with permission from ref [48].

Acoustic waves can break large droplets into microdroplets. Piezoelectric elements are often used for making low-cost acoustic wave generators for microdroplets production. Using piezoelectric transducers, Kishi et al. designed a droplet-generating system [150]. The system consisted of a compressor, a regulator, a constant pressure pump, a voltage amplifier, a high-speed camera, and an ultrasonic torsional transducer, as shown in Figure 9A. The constant pressure pump continuously pumped liquid into the device and the piezoelectric transducer generated acoustic waves at a regular interval to produce microdroplets with a uniform size. The droplet diameter is determined by the applied flow rate, the ultrasound frequency, and the radius of the nozzle. Besides, the produced droplets can be applied in noncontact deposition for liquid crystal displays, drug delivery systems, and biomedical research. Similarly, Fujimoro et al. developed a droplet production system with a torsional bolt-clamped Langevin-type transducer [151]. The two constant pressure pumps ensure that the phases of water and oil are steady and adjustable; through the transducer, droplets were generated in ambient liquid; the inset illustrated the structure of the designed transducer incorporated with a micropore plate and piezoelectric elements. This platform can generate droplets continuously as long as the pressure pump keeps injecting liquid through the transducer. The diameter of the generated water-in-oil (W/O) and silicone emulsions was 62.5 ± 2.6 µm, while the driving frequency and the vibrational velocity of micropore kept constant at 37.0 kHz and 96.5 mm/s.

Apart from aqueous microdroplets, Figure 9B illustrates an acoustic-based miniaturised system for producing LM MPs [48]. The piezoelectric transducer is stuck to the thin glass slide that adheres to the PDMS post to maximize the vibration; the Cu tape is used as electrode. The polymethylmethacrylate (PMMA) wall is attached to the thin glass slide with the 45° angle, leading to the most efficient production of EGaIn microdroplets. The acoustic waves generated from the piezoelectric transducer disk can transfer destructive forces to the droplets to compete with cohesive forces on the liquid interface, breaking it into microscale (40–50 µm) drops. This system enables the production of microdroplets with controllable size by tuning applied voltage to change the surface tension of the liquid metal and effectively avoids the use of cumbersome and expensive sonication bath or probe. Besides, such miniaturised system shows the versatility that can also be used as electrochemical sensor for heavy metal ion detection. For example, the EGaIn microdroplets coated WO_3_ NPs fabricated by the platform can detect the Pb^2+^ with the sensitivity minimizing to 500 ppb.

### 3.3. Centrifugal and Spinning Systems

**Figure 10:**
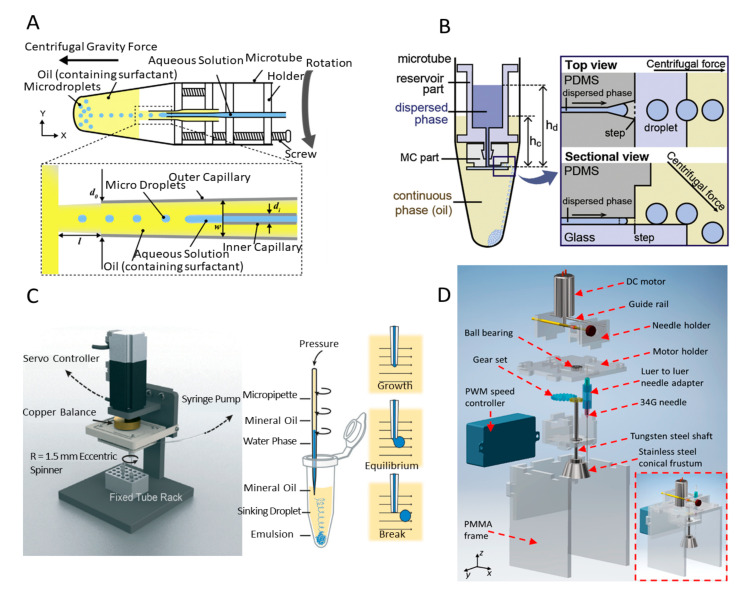
Schematics of centrifugal and spinning systems. (**A**) Illustration of the centrifuge-based axisymmetric coflowing microfluidic device. Reprinted with permission from ref [154]. (**B**) Components and schematic of the centrifuge-based step emulsion device. Reprinted with permission from ref [155]. (**C**) Schematic illustration of the liquid emulsion generator and process of droplet formation. Reprinted with permission from ref [49]. (**D**) Schematic representation of the off-chip spinning microdroplet generator. Reprinted with permission from ref [129].

Centrifugal forces can also be harnessed to break aqueous solutions into microdroplets. Centrifugal forces are generated by the rotation of liquid, and the microdroplets are pinched off from the orifice (no limitation of the number of capillary orifices) when the centrifugal force surpasses the interfacial tension exerted by the capillary orifice. Gelation of the produced sodium alginate microdroplets can be induced using calcium chloride (CaCl_2_) solution [152]. Furthermore, the shapes and morphologies of MPs can be tuned effectively by the partial dissolution of the products and manipulating the 3D microflows to deform the precursor microdroplets, realizing complex-shaped 3D multicompartmental MPs [153]. This type of devices has numerous advantages including easy fabrication, low cost, small volume required, and less sensitivity to fluid’s properties. A centrifuge-based axisymmetric coflowing microfluidic device can be prepared without the need of any photolithography process. It consists of two round capillaries (inner and outer capillaries), a capillary holder, and a sampling microtube, which can be used for producing microdroplets, as shown in Figure 10A [154]. The screw is introduced to adjust the distance between lower and upper part of the holders. By rotating the microtube, W/O microdroplets were pinched off from inner capillary; the sizes of monodisperse water microdroplets increase as the inner capillary diameters enlarge. Additionally, Shin et al. developed a centrifuge-based step emulsification device for producing microdroplets with diameters ranging from of 18 to 90 µm (CV < 2%) [155]. The device includes a reservoir part for storage of the dispersed phase, a triangular microchannel part and a step at the channel end for droplet formation, as shown in Figure 10B. Such device is put into a commercial centrifuge with a fixed angle of 45° to provide excessive pressure on the dispersed phase to infuse the dispersed phase into a microchannel. Then, the microdroplets are pinched off and stored into the microtube. With a fixed channel height, monodisperse droplets are generated under high levels of the oil phase; on the contrary, polydisperse droplets are produced under low levels of the oil phase. The high monodisperse droplets can be generated in a high centrifugal force (~>1500× *g*), which is yet to be observed in the conventional step emulsification. Moreover, under the constant aspect ratio of the microchannel (width/height) and centrifugal force, the higher the height of the microchannel is, the larger droplets can be obtained. Such droplets highlight the potential applications in biochemical reactors and food, cosmetics, and medical industries.

Other than rotating the microtube, Chen et al. designed a liquid emulsion generator to produce W/O microdroplets by moving a micropipette in a revolving manner [49]. The system consisted of a hydrophobic-coated glass micropipette, a servo motor, an eccentric wheel, a syringe, and a syringe pump (Figure 10C). The water phase flows through the micropipette under the pressure of syringe pump while it is spun by the servo controller and then breaks into uniform droplets. Due to the higher density of the droplets compared with the mineral oil, the products can smoothly sink down the bottom of the microtube. Microdroplets with diameters ranging from 25 to 230 µm (CV < 5%), which are suitable for single cell analysis, were produced by tuning the flow rate of the continuous phase and the motion velocity of the micropipette tip. However, when the platform works under the high flow rate of water phase and the high motion velocity of the micropipette, droplets with a high polydispersity will be generated. These satellite droplets are the result of high dispensing rate of water phase, and droplets near the rotation region of the tip of micropipette are smashed by the moving micropipette with a high speed. A centrifugal microchannel array droplet generating system was established for digital polymerase chain reaction (PCR) [156]. This system not only eliminates the usage of complex microfluidic devices and control systems but also greatly suppresses the loss of materials and cross-contamination. The diameter of microdroplets depends on the size of the microchannel and the centrifugal force.

To further improve the throughput of the microdroplets generation, an off-chip microdroplet generator using a spinning conical frustum was developed, as shown in Figure 10D [129]. The direct-current motor controlled by a PWM motor controller drives the tungsten-steel shaft via a gear set, and a stainless-steel conical frustrum is fixed at the end of the shaft. The diameters of microdroplets can be tuned by rotation speed, flow rate of the continuous phase, the gap between the needle tip and the surface of the frustrum, and the application of an electric field. The higher the speed the conical frustum spins at, the smaller the diameters of microdroplets obtained. After applying the electric field between the needle and the frustum, the droplet size can further decrease due to the reduction in the interfacial tension between water and oil. Apart from water droplets, LM droplets that are relatively difficult to produce in microfluidic platforms due to the high density, viscosity, and surface tension can also be generated within the corn syrup–water mixture. The LM MPs can be adopted in microelectromechanical systems, electrochemical sensors, and constructing 3D structures. Similarly, an off-chip spinning disk microdroplet production platform using submerged electrodispersion was designed to simplify the process of the production of LM microdroplets [157]. The platform consisted of a Cu electrode, a Teflon sleeve, a 34G needle, a tungsten steel shaft, and a polymethylmethacrylate (PMMA) disk. LM microdroplets with uniform sizes (<5%) were produced using the submerged electrodispersion technique. The spinning disk provided the dragging force to take the produced microdroplets away from the ground electrode and therefore avoided the undesired coalescence of droplets.

### 3.4. Jetting Systems

**Figure 11:**
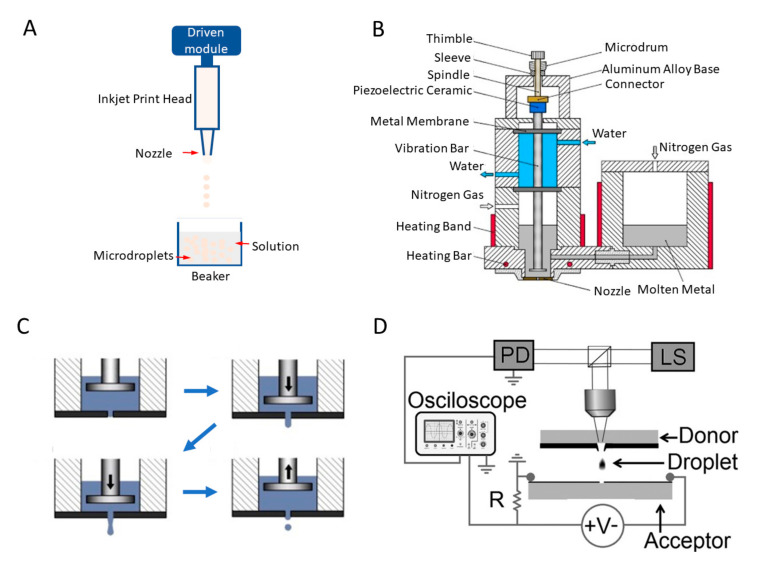
Illustrations of jetting mechanism and jetting systems. (**A**) Schematic of a typical jetting platform. (**B**) Schematic diagram of the piezoelectric membrane-piston-based jetting technology (PMPJT) system. (**C**) The principle of microdroplet formation based on the PMPJT. Reprinted with permission from ref [160]. (**D**) Schematic illustration of the aluminium (Al) microdroplets generation system combining supersonic laser-induced jetting and velocity measuring function. PD represents photodiode and LS is laser system. Reprinted with permission from ref [161].

Inkjet printing technology can rapidly create and release liquid droplets for precise deposition. In brief, jetting is induced by driving an actuator (typically pneumatic or piezoelectric) at the print head to propel the fluid, then microdroplets are expelled from the nozzle to the solution or a target surface, as shown in Figure 11A. Inkjet printing has become a standard production process in the industry and can accommodate a wide range of fluids. Numerous integrated jetting systems have been developed to address a wide variety of applications. Gao et al. presented a drop-on-demand jetting system by integrating a lift platform, a pressure control system, a syringe filter, and an imaging system [158]. With this integrated system, microdroplets with a diameter of 100 µm were made with a jetting velocity at 2.42 m/s. For the formation of microdroplets of liquid with a high viscosity, a pneumatically driven inkjet printing system was designed [50]. By assembling a pneumatic printing system for generating droplets by alternatively applying negative and positive air pressure to the precursor, a monitoring system for detecting the droplet formation process, and a measuring system for analysing the printing volume together, viscous microdroplets (1-384.5 Cp) were formed (CV of the diameter ≤1.07%).

Apart from pneumatic-driven systems, the alternating viscous and inertial forcing jetting (AVIFJ) mechanism was successfully applied in the jetting system [159]. The AVIFJ-based microdroplet deposition system contains modules of data processing, microdroplet dispensing, materials delivery, 3D motion, and observation. The piezoceramic is driven by a periodic voltage signal and moves in a reciprocating motion; the glass nozzle is fastened to the holder and the holder is connected to the piezoceramic. In the first half of the reciprocating micromotion, the fluid in the nozzle is driven by the viscous force between the fluid and the nozzle and moves downward. In the second half of the reciprocating micromotion, the fluid keeps moving downward by inertia, while the nozzle moves upward. Consequently, production of microdroplets can be achieved using the reciprocating micromotion of the nozzle. The system can form microdroplets (diameter of 52–72 µm, with a nozzle diameter of 45 µm and jetting speed of 0.4–2.0 m/s), which can be applied in addictive manufacturing and cell printing.

In addition to water, solder droplets that are suitable for metal additive manufacturing can be formed using this jetting technology. Ming et al. presented a piezoelectric membrane-piston-based jetting technology (PMPJT) system, as shown in Figure 11B [160]. A computer numerical control system is introduced to produce an electrical pulse signal that excites the piezoelectric ceramic, forcing the vibration bar to move. Figure 11C simply illustrates the working principle of PMPJT. When the electrical pulse signal applies, the vibration bar moves downward to stretch the metal membranes and some molten metal flows through the orifice of the nozzle to form a stream. After turning off the pulse signal, the membranes begin to restore and induce the shrinking of the stream at the orifice. Due to gravity and inertia of the stream, molten metal is finally separated from the main body. The spherical microdroplets are finally formed because of the surface tension. By adjusting the voltage and length of the electrical pulses and temperature of the metal, the diameter of microdroplets formed in this system ranged from around 85 to 121 µm. The system can operate at a working temperature higher than 180 °C to generate solder MPs. Similarly, StarJet technology was used to generate Al alloy MPs [126]. The assembly of the StarJet printhead includes the inserted Macor reservoir, the cap, and the body (made from Inconel 718 alloy). The reservoir outlet tube with diameter of 400–600 μm prevents the melting metal from flowing out of the printhead by counteracting capillary forces, and the nozzle chip is directly mounted under the reservoir outlet tube. The melting metal is pneumatically pushed towards the nozzle chip and forms microdroplets. Such a prototype of printhead in drop-on-demand mode enables the direct printing MPs of Al alloys, which extends to the application of Al alloy MPs in additive manufacturing. Moreover, using the principle of laser-induced forward transfer, Zenou et al. utilised a laser system to induce Al microdroplets jetting from donor to accepter, as shown in Figure 11E [161]. By measuring the electrical signal obtained once the Al microdroplet touches the accepter, the velocity of Al microdroplets can be estimated. Such a jetting process demonstrates the effective energy transfer from the laser to the Al microdroplets. Above-introduced integrated systems for synthesising MPs are summarised in Table 4.

## 4. Conclusions and Outlook

In this review, we have discussed the modular and integrated systems developed in recent years for producing a wide variety of NPs and MPs. For the synthesis of NPs, we have elucidated some representative systems based on top-down and bottom-up methods, which are summarised in the Table 2 and Table 3, respectively. In terms of top-down approaches, the products can be synthesized rapidly by breaking the bulk materials into nanoscales. Sonication probe systems are suitable for producing LM NPs with small enough sizes, and the production performance can be optimised by incorporating appropriate control modules. However, it is still problematic for producing uniform-sized NPs as the disruptive force is uncontrollable. Laser ablation systems are ideal for the production of pure metal NPs like Au and Ag. Besides, these systems are capable of fabricating specific structures of NPs by combining external fields. Because industrial scale of production requires high power and multiple sets of expensive laser systems, the above-mentioned techniques are yet to be readily used in industry.

As for bottom-up approaches for producing NPs, microfluidics can precisely control the reaction time and volume to enable various NPs production with uniform and monodisperse sizes. In recent years, different groups had designed various structures of microfluidic chips and integrated multiple synthetic procedures into a single microfluidic device to produce NPs with desired functions and morphologies. Moreover, multichannel microreactor systems for providing continuous flow enable the scaling up of production, which would allow for expansion to achieve an industrial level of production. Nevertheless, the fabrication of microfluidic chips requires the photolithography techniques and specialised microfluidic facilities in cleanroom, which limits accessibility. In addition, the integrated systems usually need to be driven by external pumping systems—an inconvenience for settings other than laboratory. In addition to microfluidics, flame synthesis is a powerful bottom-up method. It does not need tedious steps and can easily form metastable compositions with a high purity in a short reaction time. However, such systems still face challenges. For example, it is difficult to produce more complicated NPs other than noble metals and carbon using flame synthesis platforms; also, the properties of substrate limit the productions and utilisation of NPs.

For the production of MPs (summarized in Table 4), microfluidic technology has many advantages, including the ability to control size, morphology, and composition of particles. In recent years, 3D printing technology further facilitates the fabrication of microfluidic devices without the need of photolithography. This technology lowers the entry requirements of using microfluidics and benefits the advancement of future integrated systems. In addition, other MPs generation systems have been developed using techniques including acoustic, centrifugal, spinning force, and jetting. By controlling parameters such as acoustic frequency, rotating speed, and driving force/frequency, MPs with various sizes, morphologies, and compositions, can be synthesized on demand. These integrated systems avoid complicated structures and reduce the difficulties of operation, which may lead to an expansion of the applications of MPs.

On the other hand, the development of modular and integrated systems for the versatile production of NPs/MPs await enhancement. For instance, some integrated systems require large space and hard to be decomposed into modules. In addition, in many cases, bulky peripherals are still needed to operate and control the platforms. Besides, the use of specialised and noncommercial components in many of the systems limits their accessibility. Regardless of these hardships, we still believe that further development of modular and integrated systems will bridge the gap between harnessing the vast potential of NPs/MPs research and the difficulty of synthesising NPs/MPs in simple, repeatable, and on-demand manners in different laboratories. We anticipate that researchers can readily select and establish synthesis systems for the production of customised NPs/MPs with a simplified process, thereby breaking the obstacle in exploring a wider range of applications of NPs/MPs. We envisage that some modular and integrated systems possess the potential to meet the industrial scale and quality, making it possible to further reduce the cost and complexity of synthesising NPs/MPs. As such, assisting the production of NPs and MPs using modular and integrated systems with the characteristics of automatic control, simple user interface, customisable functions, which only requires basic equipment which most of the laboratories can offer, can certainly facilitate interdisciplinary innovations for unleashing a wider range of research directions.

## Figures and Tables

**Table 1:** Overview of applications for different types of nanoparticles (NPs).

Type to NPs	Applications	Reference
Liquid metal (EGaIn, Galinstan, other Ga-based alloys)	Soft/flexible/wearable electronics Biomedical applications (e.g., drug delivery, medical imaging, therapeutics, and antimicrobial activities)	[12,14,15,16]
Nobel metal (Au, Ag, platinum group of metals)	Antimicrobial activities Optoelectronics Catalysis Biomedical applications (e.g., drug delivery, medical imaging, and photothermal therapeutics)	[17,18,19,20]
Transition metal (Cu, Ni, etc.)	Wastewater treatment Antimicrobial and anticancer activities Catalysis Biomedicine Energy storage	[21,22,23,24]
Oxides of metals (Fe_2_O_3_, SnO_2_, Al_2_O_3_, etc.)	Anti-infective applications Electrochemical sensing and biosensing Catalysis Optoelectronics Medical imaging	[25,26,27]
Semiconductor quantum dots (CdSe, CdTe, CdSeTe, etc.)	Catalysis Solar concentrators Medical imaging Cellular imaging	[28,29,30,31]
Carbon-based materials (CNTs, graphene, CB, etc.)	Electrochemical sensing Energy storage Catalysis Cellular imaging Biomedical applications (e.g., bioimaging, biosensors, drug delivery, theranostics, and tissue engineering)	[4,32,33,34,35]
Organic polymer (PLGA, PLGA@HF, PEG, etc.)	Mostly biomedical applications (e.g., drug delivery, tissue regeneration, molecular imaging, and cancer phototherapy)	[36,37,38]

**Table 2:** Overview of integrated systems for the top-down production of nanoparticles (NPs).

NPs Type	Enabling Technologies/Modules	Crucial Parameters	NP Size (nm)	Costs ^1^	Year	Reference
EGaIn	Microfluidics Ultrasonic bath	Dimension of microchannel s Centrifugal force	200–700 (peak)	★★	2018	Tang [51]
EGaIn	Liquid-based nebulization	Input voltage	~160–200	★	2019	Tang [55]
EGaIn additive	Ultrasonic bath Cooling water machine		286 ± 21	★★★	2020	Guo [74]
Au	Laser ablation	Subpulse number in an envelope	~4–120	★★★	2017	Yu [62]
Ag	Laser ablation	Liquid medium	3.4–15.4	★★★	2020	Menazea [66]
Au, Ag	Laser ablation Ultrasonic bath	Ultrasonic field	5.4–7.8 (Au)/7.9–12.1 (Ag)	★★★★	2020	Hu [59]
Au	Laser ablation	pH	13 ± 3	★★★	2017	Palazzo [75]
Au	Laser ablation		14 ± 2.1	★★★	2015	Affandi [76]
Au	Laser ablation Magnetic field	Field tensity	~3–8	★★★★	2016	Serkov [68]
Au	Laser ablation Magnetic field	Residence time in the external magnetic field	~20	★★★★	2019	Shafeev [69]
Ag	Laser ablation	Laser pulse energy	~10	★★★	2015	Valverde-Alva [77]
Au	Laser ablation	Laser fluence Liquid media	~3.16 (average)	★★★	2015	Tomko [78]
Ag	Laser ablation	Laser wavelength	3 and 20	★★★	2016	Kőrösi [64]
Ag, Cu, Ag-Cu alloy	Femtosecond laser ablation Laser irradiation		~33.4(Ag)/~22.7(Cu)/~23.8(Ag-Cu alloy)	★★★	2019	Bharati [79]
Copper (I and II) oxide	Continuous flow Laser ablation		~14	★★★	2019	Al-Antaki [80]
Pt, Au, CuO	High-speed pulsed laser ablation	Laser fluences Repetition rates Ablation time	4–7	★★★	2016	Streubel [73]
Al, Ti	Laser ablation	Laser pulse number Water depth	19–38 (Ti)/29–41 (Al)	★★★	2015	Mahdieh [63]
Pt, Au, Ag, Al, Cu, Ti	Laser ablation Two scanning systems	Repetition rate of laser	7	★★★★	2016	Streubel [61]
CuO	Laser ablation in liquid	Laser energy	3–40	★★★	2016	Khashan [81]
Cu_3_Mo_2_O_9_ nanorods	Laser ablation Electrochemistry		~100 (diameter) ~3 μm (length)	★★★	2011	Liu [70]
CdO	Pulsed laser ablation		~47	★★★	2017	Mostafa [82]
Au@CdO	Pulsed laser ablation		~11.35	★★★	2017	Mostafa [83]
Transition metal vanadates nanostructures	Laser ablation Electrochemistry	Applied voltage	~300 (diameter) ~100–140 (thickness)	★★★	2012	Liang [72]
Cobalt oxide/hydroxide	Laser ablation	Laser wavelength Laser fluence	~10–22 (average)	★★★	2014	Hu [84]
CeO_2_/Pd	Pulse laser ablation		~20(CeO_2_)/~9(Pd)	★★★	2015	Ma [85]
GeO2 nanotubes/spindles	Laser ablation Electrical field Ultrasonic vibrator	Applied electrical field	~200–500 (nanotube) ~200–400 (spindle)	★★★★	2008	Liu [67]
FePO_4_	Ultrasonic intensification Impinging jet reactor	Ultrasonic power	107–191	★★★★	2019	Guo [86]
α-Fe_2_O_3_	laser ablation	Laser fluencies	50–110	★★★	2015	Ismail [87]
Fe_2_O_3_	Laser ablation/fragmentation technique	Liquid media	50–200	★★★	2014	Pandey [88]
Magnetic NPs	Laser ablation Magnetic field		~200–500	★★★★	2014	Liang [60]
Carbon nanotube	Laser ablation	Laser wavelength	1.3	★★★	2015	Chrzanowska [89]
Carbon	Pulsed laser ablation in vacuum		~33	★★★	2017	Kazemizadeh [90]

^1^ The number of asterisks (★) represents the cost of synthesis system; 1 means relatively low cost, while 5 means expensive.

**Table 3:** Overview of integrated systems for bottom-up methods.

NPs Type	Enabling Technologies/Modules	Crucial Parameters	NP Size (nm)	Costs ^1^	Year	Reference
CdSe	Continuous-microflow synthesis High-pressure microreactor	Solvent phase Concentration Temperature Residence time	~3–6	★★★★	2008	Marre [94]
CdSe	Combinational microreactors	Temperature Reaction time Reaction additive concentration	~2–4.5	★★★★	2010	Toyota [95]
CdTe, CdSe, alloy CdSeTe	Multichannel droplet reactor			★★★★	2013	Nightingale [91]
InP/ZnSeS	Millifluidic reactor system	Flow rate Reactor temperature Shell precursor concentration	5.9 ± 1.2	★★★	2018	Vikram [96]
PbS	Droplet-based microfluidic	Temperature Flow rate	2–6	★★	2015	Lignos [97]
Au	Millifluidic benchtop reactor system “Y” mixer Flow synthesis	Concentration	~2–40	★★★	2013	Lohse [100]
Au	Zigzag micromixer	Seeds volume Residence channel length	75 ± 6	★★★	2017	Thiele [117]
Au, bimetallic AuPd	Millifluidics Continuous flow	Flow rate Reactor geometry	6.4 ± 1.5 (I-shape connection)/6.3 ± 1.3 (helical reactor)	★★	2019	Cattaneo [102]
Ag	Droplet-based microfluidic reactor	Static mixing Temperature Flow rate	4.37–11.45	★★★	2018	Kwak [104]
Ag	Drop-based microfluidics	Concentration ratios Flow rates	4.9 ± 1.2	★★	2016	Xu [118]
Nobel metal	Millilitre-sized droplet reactors	Capping agent Reductant Reaction temperature	~9–50	★★★	2014	Zhang [101]
Nobel metal	Multichannel droplet reactor	Capping agent Reductant Reaction temperature	~2.5	★★★	2018	Niu [105]
Pd-Pt, Pd@Au (core@shell)	Duo-microreactor	Concentration	18.0 ± 2.7	★★★★	2019	Santana [119]
BaSO_4_, Au, CaCO_3_	Segmented flow microchannel Passive picoinjection	Injection volume	30–40 (BaSO_4_) 32–91 (Au)	★★★★	2018	Du [120]
Superparamagnetic iron oxide	Micellar electrospray		36 ± 6	★★★★	2014	Duong [121]
Ni	Continuous flow microreactor	Flow rates	~6.43–8.76	★★★	2015	Xu [122]
Fe_3_O_4_	Flow synthesis “T” mixer	Linear velocity Residence time Reactor dimension	4.9 ± 0.7	★★	2015	Jiao [123]
PLGA@HF, PLGA@AcDX	Multiplex microfluidics	Flow rates	~60–550	★★	2017	Liu [108]
PLGA	Microfluidic origami chip	Flow rates	~100	★★	2013	Sun [106]
PLGA, hydrophobic chitosan, acetylated dextran	3D coaxial flows Glass capillaries		~100–400	★★★	2015	Liu [107]
Metal-organic frameworks (MIL-88B)	Nanolitre continuous reactor Segmented flow	Residence time Temperature Volume slug	90–900	★★★	2013	Paseta [124]
Silica, polymersomes, niosomes	Microreaction technology	Flow rates	238–361 (silica)/275–75 (niosomes)	★★★	2019	Bomhard [125]
Ag	Liquid flame spray	Passing times	~10–100	★★★	2017	Brobbey [92]
SnO_2_	Single droplet combustion Flame spray pyrolysis	Metal-precursor concentration	~3–39	★★★★	2020	Li [112]
α-Al_2_O_3_	Flame synthesis	Ratios of oxygen and acetyl	50–150	★★★	2014	Kathirvel [114]
Cs_0.32_WO_3_	Flame-assisted spray pyrolysis	Flame temperature Flow rate	~6–300	★★★	2018	Hirano [116]
Fe/Al_2_O_3_	Flame spray pyrolysis	Precursor molar ratio Multicomponent structures	183–187	★★★	2016	Hafshejani [115]
Carbon	Flame synthesis Conical chimney	Combustion regime	~200	★★★	2016	Esmeryan [113]
Carbon nanotube	Flame synthesis Methane diffusion flames	Sampling time Sampling height Sampling substrate	30–110	★★★	2018	Chu [111]
Onion-like carbon	“Wick-and-oil” flame synthesis		~25 ± 5	★★★	2016	Mohapatra [93]

^1^ The number of asterisks (★) represents the cost of synthesis system; 1 means relatively low cost, while 5 means expensive.

**Table 4:** Overview of integrated systems for synthesis of microparticles (MPs.)

MPs Type	Enabling Technologies/Modules	Crucial Parameters	MP Size (μm)	Costs ^1^	Year	Reference
PEDOT/PSS-agarose hybrid MPs	Microfluidic droplet generator	Continuous oil flow rate	20–80	★★	2016	Lee [127]
Solid core enzyme-immobilised microcapsules	Flow focusing		580 ± 10	★★	2019	Varshney [132]
Magnetic droplets	Step emulsion device Magnetically driven microfluidic droplet generation technique	Dimensions of channels	85–125	★★	2016	Kahkeshani [130]
W/O emulsions W/O/W emulsions	Flow focusing Droplet-based microfluidics Commercially available self-setting rubber	Flow rate Nozzle diameter	100–500	★	2015	Lapierre [131]
Chitosan microspheres	512-microchannel geometrical passive breakup device T-junction	Flow rate	40.0 ± 2.2	★★	2019	Kim [133]
PLGA microspheres	512-channel geometric droplet-splitting microfluidic device 256 T-junction		6.56	★★	2020	Kim [136]
Cell-laden microgel	Flow-focusing platform On-chip	Cell concentration	~240–300	★★	2019	Mohamed [138]
Drops	Parallelised microfluidic device Millipede device	Device geometry	20–160	★★	2016	Amstad [135]
Free-floating polymer (PEGDA)	Contact flow lithography system	Microchannel dimensions	20–150	★★★	2015	Goff [139]
W/O and O/W emulsions	Glass microfluidic device Step emulsification		80.9 (CV = 2.8%)	★★	2017	Ofner [137]
Chitosan/TiO_2_ composite	Factory-on-chip Modularised microfluidic reactors		539.65	★★★	2017	Han [134]
Water-in-water (W/W) emulsions	Microneedle-assistance Microfluidics Flow focusing	Column pressure	5–65	★★★	2019	Jeyhani [128]
W/O emulsions	Electrical detection Microfluidics Closed-loop control	Flow rate	200	★★★★	2017	Fu [144]
Liquid metal	Microfluidic flow-focusing device	Electrical potential Flow rate	~80–160	★★★	2016	Tang [48]
W/O and oil-in water (O/W) emulsions	3D-printed droplet generator Plug-and-play	Liquid flow rate ratio Viscosity of the dispersed phase	~50	★★	2016	Zhang [146]
PEGDA	3D-printed generator Screw-and-nut	T-junction gap height Flow rates	34–1404	★★	2019	Nguyen [147]
W/O droplets	3D-printing technology Millifluidics Chimney-shaped void geometry	Flow rates Apex angle	36–616	★★	2019	Hwang [148]
Magnetic liquid metal	3D-printed coaxial microfluidic device	Orifice diameter Flow rate ratio	650–1900	★★★	2020	He [149]
EGaIn	Acoustic waves Electrochemistry Electrocapillary	Oxidative/reducing voltages Activating frequency	10–80	★★★	2016	Tang [48]
Water-in-oil (W/O) emulsions	Ultrasonic transducer	Vibrational velocity Pressure	62.5 ± 2.6	★★★	2018	Fujimoro [151]
Pure water, silicone oils	Ultrasonic torsional transducer	Pressure Resonance frequency Diameter of liquid column	~80–120	★★★	2015	Kishi [150]
W/O microdroplets	Glass-capillary-based microfluidic device Tabletop minicentrifuge	Diameter of inner and outer capillary orifice	~6.6–13.8	★★	2014	Yamashita [154]
W/O emulsions	Spinning micropipette liquid emulsion generator	Flow rate Motion velocity of the micropipette	25–230	★★	2016	Chen [49]
W/O emulsion	Centrifugal microchannel	Size of microchannels Centrifugal force	~52.5	★★	2017	Chen [156]
Calcium alginate	Centrifugal microfluidic technique	Centrifugal force Circumference of the channel outlet	~109–269	★★★	2015	Liu [162]
W/O picolitre droplets	Centrifuge-based step emulsification device	Level of oil phase Centrifugal force Height of microchannel	18–90	★★★	2019	Shin [155]
Gallium-based liquid metal	Submerged electrodispersion technique Spinning disk	Electric field Flow rate Rotation speed of the disk	~10–800	★★★	2019	Zhang [157]
Water, liquid metal, hydrogel, double emulsions	Spinning conical frustum	Rotational speed Applied voltage Flow rate	~200–550	★	2019	Tang [129]
Sodium alginate multicompartmental particles	Centrifuge-based droplet shooting device	Barrel configuration Diameter of capillary orifice	99 and 16	★★★	2012	Maeda [152]
Sodium alginate with complex shape	Centrifuge 3D nonequilibrium-induced microflows	Diffusional flow Marangoni microflows	~112.4–135.1 (various shapes)	★★★	2016	Hayakawa [153]
Janus MPs	Centrifugal gravity UV irradiation		282 (mean)	★★★	2020	Tsuchiya [163]
Solder (Sn63Pb37)	Piezoelectric membrane-piston-based jetting technology	Pulse length Voltage value Temperature	~85	★★★★	2019	Ma [160]
PDMS, UV-curing optical glue (high viscosity >2000 cps)	Tip-assisted electric field intensity enhancement effect High-resolution capability of EHD printing	Applied voltage Gap distance Nozzle inner diameter Deposition time	>2.3	★★★★	2019	Zou [164]
Al	Pneumatic drop-on-demand technology	The aspect ratio of the nozzle hole The distance between inlet hole and nozzle hole	359.9	★★★★	2017	Zhong [165]
Ink drops	Pneumatic valve Feedback control Ejection technology Machine vision	Solenoid valve “ON” time		★★★★★	2018	Wang [166]
Al alloys (AlSi12)	StarJet technology	Applied pressures	235 ± 15	★★★★	2017	Gerdes [126]
Alginate	Drop-on-demand jetting Piezoelectric print-head	Voltage waveform Microdroplet velocity Concentration of CaCl_2_ solution	~80–110	★★★★	2016	Gao [158]
Water drops	Piezo-actuated microdroplet generator Drop on demand	Deflection voltage Suction and compression time Nozzle diameter	450–1000	★★★★	2014	Sadeghian [167]
Chitosan aerogel	Jet cutting Supercritical drying of gel	Nozzle diameter Cutting disc velocity Number of wires of the cutting disc	700–900	★★★	2020	López-Iglesias [168]
Sodium alginate	Alternating viscous and inertial force jetting mechanism	Applied voltage Nozzle diameter Fluid viscosity	~30–80	★★★★	2017	Zhao [169]
Sodium alginate	Alternating viscous and inertial force jetting mechanism	Actuation signal waveforms Nozzle dimensional features Solution velocity	53–72	★★★★	2015	Zhao [159]
Al	Supersonic laser-induced jetting	Incubation time Droplet velocity	~3.9	★★★★	2015	Zenou [161]
High viscous microdroplets	Pneumatically driven inkjet printing system	Droplet volume Standoff distance frequency	~143–247 (12.2–63.5 nL)	★★★★	2016	Choi [50]

^1^ The number of asterisks (★) represents the cost of synthesis system; 1 means relatively low cost, while 5 means expensive.
